# Supplementary material for: The causal relationship between the human gut microbiota and pyogenic arthritis: a Mendelian randomization study
Source: Front Cell Infect Microbiol. 2024 Nov 26;14:1452480. doi: 10.3389/fcimb.2024.1452480 (PMC11629706; doi:10.3389/fcimb.2024.1452480)
Supplement: Supplementary file 1 [file DataSheet1.zip › mendelian test/ebi-a-GCST90027824.csv_forest.pdf]

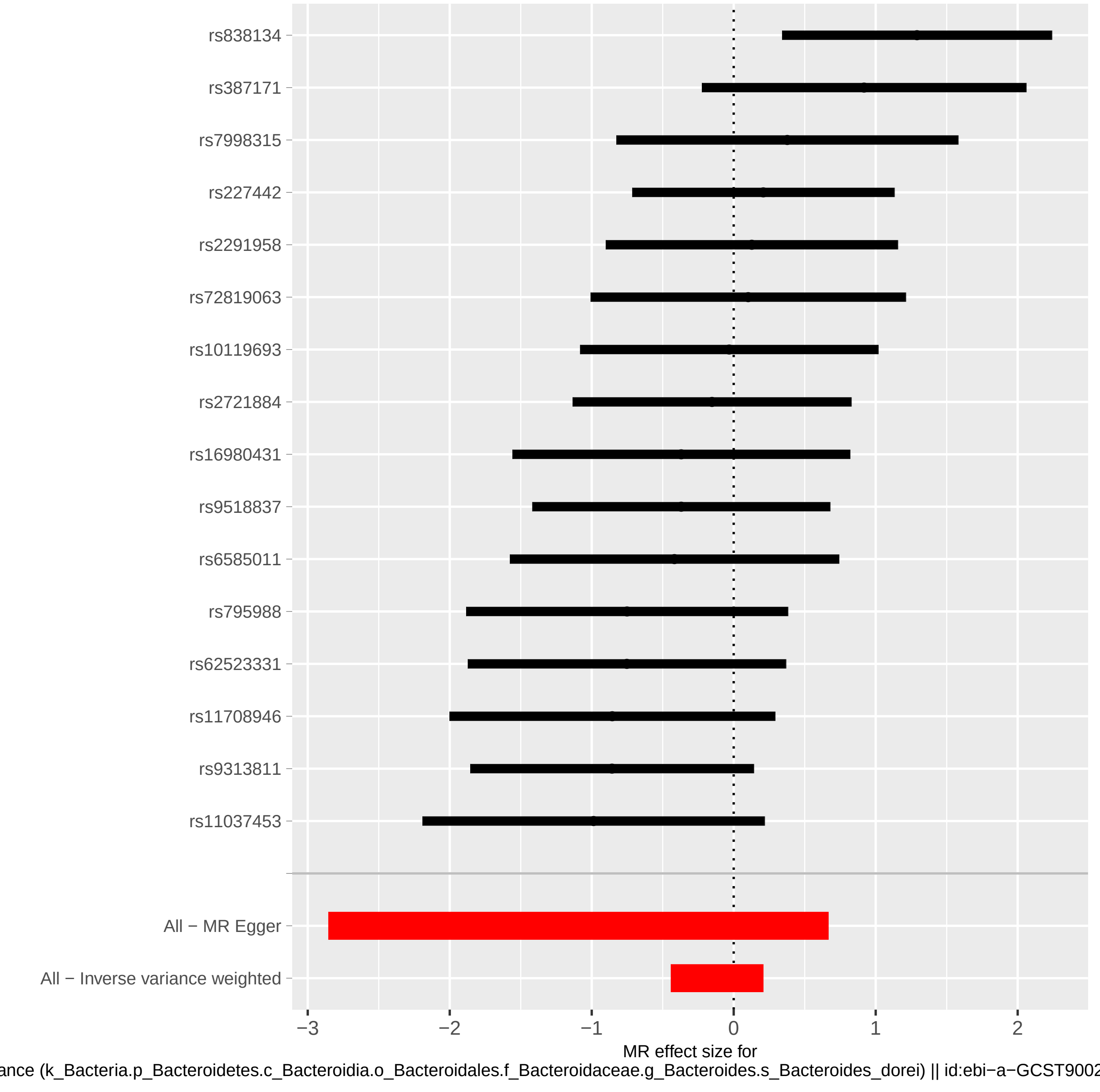

rs838134
rs387171
rs7998315
rs227442
rs2291958
rs72819063
rs10119693
rs2721884
rs16980431
rs9518837
rs6585011
rs795988
rs62523331
rs11708946
rs9313811
rs11037453
All − MR Egger
All − Inverse variance weighted
−3
−2
−1
0
1
2
MR effect size for
ance (k_Bacteria.p_Bacteroidetes.c_Bacteroidia.o_Bacteroidales.f_Bacteroidaceae.g_Bacteroides.s_Bacteroides_dorei) || id:ebi−a−GCST9002
